# A cross‐sectional study of anesthesia medical staff's occupational and health status in nongovernment medical institutions in China

**DOI:** 10.1111/jep.14194

**Published:** 2024-11-08

**Authors:** Bo Wang, Kunpeng Liu, Hui Shi, Xuanling Chen, Xuewei Qin, Lan Yao, Yongxing Sun, Wei Chai, Chunhong Liu

**Affiliations:** ^1^ Department of Anesthesiology Peking University International Hospital Beijing China; ^2^ Department of Anesthesiology Shijingshan Teaching Hospital of Capital Medical University, Beijing Shijingshan Hospital Beijing China; ^3^ Department of Anesthesiology Sanbo Brain Hospital, Capital Medical University Beijing China; ^4^ Department of Anesthesiology Xi'an International Medical Center Hospital Shaanxi China; ^5^ Department of Anesthesiology Huainan Chaoyang Hospital Anhui China

**Keywords:** anesthesiologist, health status, Nongovernment medical institution, nurse anesthetist, occupational status

## Abstract

**Background:**

Nongovernment medical institutions have gradually become a significant part of China's healthcare system, with a growing numbers of staff. However, the current status of anesthesiology staff in these institutions is unclear. To gain insight into this situation and to compare it with public hospitals, the national anesthesia professional committee of the Chinese Nongovernment Medical Institutions Association (CNMIA) designed and conducted the national cross‐sectional survey.

**Methods:**

We conducted a national cross‐sectional study to investigate the occupational and health status of anesthesiology staff in Nongovernment medical institutions. Additional questions were included for the directors of the anesthesiology department to understand their work stress and the reasons for employee turnover. The electronic questionnaire was created using Questionnaire Star and distributed by Anesthesia Professional Committee of the CNMIA through the QR code links and WeChat.

**Results:**

A total of 1111 questionnaires were collected, including 989 from anesthesiologists and 122 from nurse anesthetists. The overall job satisfaction score (MSQ) was 75.57 ± 12.32 and the average fatigue score (MFI‐20) was 49.10 ± 10.90. High‐risk factors for severe fatigue included being aged 31–40, holding a middle title, frequently working night shifts, having long working hours, and participating surgeries classified as ASA III or higher. The most common disease reported was difficulty falling asleep/insomnia. Multivariate logistic regression analysis showed that men (OR = 0.662, 95% CI: 0.482–0.909, *p* < 0.05), those with a bachelor's degree (OR = 2.152, 95% CI: 1.186–3.903, *p* < 0.05), individuals with heavy workloads (OR = 2.999, 95% CI: 1.493–6.024, *p* < 0.01), poor health (OR = 4.280, 95% CI: 1.216–15.057, *p* < 0.05), and high MFI‐20 scores (OR = 1.085, 95% CI: 1.067–1.103, *p* < 0.001) were more likely to suffer from insomnia. Directors identified medical quality and safety management as their main source of stress and low income as the primary reason for employee resignation.

**Conclusions:**

Nongovernment medical institutions have fewer employees, similar workloads, relatively low job stress and higher job satisfaction compared to public hospitals. Low income and difficulty falling asleep/insomnia are significant issues that require attention.

## 1 BACKGROUND

China's healthcare system is currently government‐led and assisted by the market, with hospitals comprising both public and Nongovernment(private) institutions. By the end of 2022, there were 25,230 Nongovernment hospitals nationwide, accounting for 68.2% of all hospitals. However, public hospitals comprised 70.0% of hospital beds compared to 30.0% in Nongovernment hospitals. At the same time, the number of medical practitioners was 5.72 million in public hospitals and 1.64 million in Nongovernment hospitals, with a ratio of 77.8% and 22.2%, respectively. Despite rapid development, Nongovernment medical institutions still face issues such as relatively small scale and weaker service capabilities. In 2022, public hospitals in China saw 3.19 billion outpatient and emergency visits, accounting for 83.4% of the total hospital visits, while Nongovernment hospitals had 630 million outpatient and emergency visits, representing 16.6% of the total hospital visits (www.gov.cn).

The significant improvement in healthcare quality and the rapid increase in the number of surgeries symbolize the fast‐paced advancement of modern medicine in China over the past decades. Simultaneously, the field of anesthesiology in China has undergone significant changes and continuous growth, transitioning from traditional Chinese medicine anesthesia to modern anesthesia.^1^ In 2018, the first comprehensive survey of all anesthesiology departments in Chinese mainland hospitals was conducted to collect data on the current status of anesthesiology in China.^2^ This survey included aspects such as the autonomous establishment of anesthesiology and their influencing factors, the proportion and geographic distribution of anesthesiologist human resources, the annual workload of anesthesiologists, and the relationship between the proportion of anesthesiologists and health economic indicators compared with other countries. However, no similar examination has been done for Nongovernment hospitals.

The differences between Nongovernment and public hospitals stem from varying levels of government and social support, as well as the unequal distribution of medical resources. These disparities include financial resources, equipment, recognition, patient sources, occupational statuses, and health conditions. To better understand the current situation of anesthesia staff in Nongovernment medical institutions in China, promote the development of Nongovernment medical institutions, and comprehend the differences between them and public hospitals, the national anesthesia professional committee of the Chinese Association of Nongovernment Medical Institutions (CANMI) designed and conducted a national cross‐sectional survey. As the leading national and professional organization for Nongovernment healthcare in China, CANMI aims to assess the occupational and health status of medical staff to promote the growth of these institutions and protect healthcare workers' well‐being. This study was the first comprehensive survey of anesthesia departments in Nongovernment hospitals in mainland China.

## 2 METHODS

The nationwide survey was conducted from August 1 and December 31, 2022. All Nongovernment hospital providing anesthesia care in mainland China were included, and the potential respondents were healthcare practitioners in anesthesia departments.

### 2.1 Questionnaire design

The questionnaire was designed to be completed by either the anesthesiologist or the nurse anesthetist in each anesthesia department at Nongovernment hospitals. To encourage as many anesthesiologists and nurses from Nongovernment hospitals to participate in our survey as possible, an electronic version of the questionnaire was developed using Questionnaire Star and distributed via QR code links and WeChat by the Anesthesia Professional Committee of CANMI. This organization is currently the most recognized, influential, and largest among Nongovernment hospitals in mainland China. Additionally, WeChat, being one of the most widely used social media platforms, ensured the accessibility of the survey. Furthermore, department directors encouraged the medical staff to participate, and participants were asked to share the questionnaire across various WeChat groups for healthcare professionals, which helped reduce nonresponse bias. The validity and reliability of the survey questionnaire were evaluated and endorsed by experts from CANMI and independent statisticians. The questionnaire comprised four parts with a total of 38 items. To ensure comprehensive data collection, all questions were marked as mandatory. Each WeChat account was restricted to one submission to prevent duplication. The survey was administered anonymously to safeguard participants' privacy. Considering the questionnaire's nature, the ethics committee deemed it unnecessary for respondents to provide informed consent.

### 2.2 Questionnaire content

Part 1 collected general information, including gender, age, marital and family status, professional title, position, education, income, and years of work. It also includes details about the type, level, and location of the hospital.

Part 2 investigated the work intensity, scientific research and teaching involvement of medical staff. This included night shift frequency, work hours, number of surgical cases, proportion of ASA level III and above patients, perceived workload, participation in scientific research and teaching activities, and number of published papers.

Part 3 assessed job satisfaction, self‐fulfillment, and professional identity of medical staff. We utilized the Minnesota Satisfaction Questionnaire (MSQ) to assess medical staff satisfaction with their current work situation.

Part 4 utilized the 12‐item Short‐Form Health Survey (SF‐12) to evaluate quality of life, the Multidimensional Fatigue Inventory (MFI) to measure work‐related fatigue, and the Insomnia Severity Index (ISI) to assess sleep quality. It also included inquiries about current illness.

Part 5 was specifically for heads of anesthesia departments. This section investigated the number of anesthesia staff, issues such as staff shortages and excessive fatigue, reasons for employee turnover, and sources of pressure on the department heads. If the respondent was not the head of the anesthesia department, this section will be automatically skipped.

### 2.3 Statistical analysis

SPSS version 22.0 was used for the statistical analysis of the survey results. Measurement data are presented as the mean ± standard deviation. For normally distributed data with homogeneous variances, one‐way analysis of variance (ANOVA) was utilized; otherwise, the rank sum test was applied. Descriptive and categorical data were expressed as proportions, and statistical inference utilized chi‐squared or Fisher's exact probability tests. To explore the relationship between insomnia and demographic factors, as well as occupational status, multivariate logistic regression analysis was conducted. *p* < 0.05 was considered statistically significant.

## 3 RESULTS

**Figure 1:**
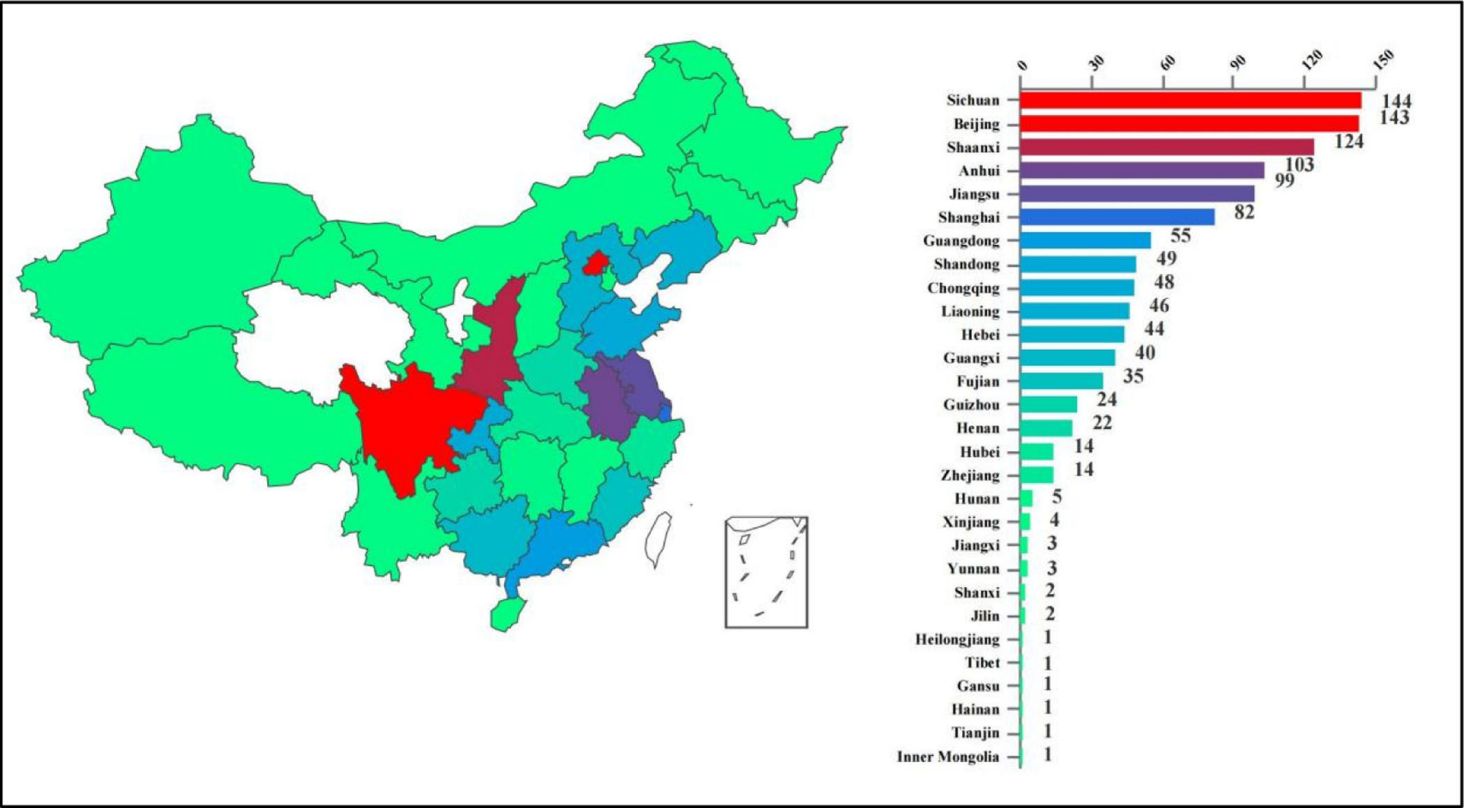
Distribution of survey questionnaires by province. This figure illustrates the geographic distribution of survey questionnaires across different provinces. The data highlights how many questionnaires were collected from each province, providing insights into the regional representation within the survey.

A total of 1111 questionnaires were collected, with the geographical distribution shown in Figure 1. Among the respondents, 989 were anesthesiologists and 122 nurse anesthetists.

### 3.1 General information

Most anesthesiologists are male, aged between 31 and 50, with over 15 years of work experience. In contrast, nurse anesthetists are predominantly female, relatively younger, and have less than 10 years of work experience. 85.3% of nurse anesthetists have an annual income of 150,000 or less. Although there are more anesthesiologists with an annual income above 150,000, the majority still earn 150,000 or less. Detailed information can be found in Table 1.

**Table 1:** The demographics of participants (*n* = 1111).

Variables	Anesthesiologist (*n* = 989)	Nurse (*n* = 122)
Gender		
Male	615 (62.2%)	20 (16.4%)
Female	374 (37.8%)	102 (83.6%)
Age (years)		
≤30	129 (13.0%)	56 (45.9%)
31–40	339 (34.3%)	58 (47.5%)
41–50	362 (36.6%)	6 (4.9%)
50	159 (16.1%)	2 (1.6%)
Marital status		
Single/divorced/widowed	128 (12.9%)	50 (41.0%)
Married without children	60 (6.1%)	8 (6.6%)
Married with children	801 (81.0%)	64 (52.5%)
Education		
Junior college or below	61 (6.2%)	33 (27.1%)
Bachelor's degree	750 (75.8%)	88 (72.1%)
Master's degree	133 (13.5%)	1 (0.8%)
Doctoral degree	45 (4.6%)	0 (0%)
The intention to improve degree		
No	398 (42.2%)	35 (28.7%)
Yes	546 (57.8%)	87 (71.3%)
Staff title		
Ungraded or Junior	181 (18.3%)	72 (59.0%)
Middle	392 (39.6%)	48 (39.3%)
Sub‐senior	299 (30.2%)	1 (0.8%)
Senior	117 (11.8%)	1 (0.8%)
Annual income (ten thousand RMB)		
≤15	386 (39.0%)	104 (85.3%)
16–20	217 (21.9%)	13 (10.7%)
21–25	129 (13.0%)	3 (2.5%)
26–30	106 (10.7%)	0 (0%)
31–35	55 (5.6%)	0 (0%)
>35	96 (9.7%)	2 (1.6%)
Type of hospital		
General	682 (69.0%)	93 (76.2%)
Special	307 (31.0%)	29 (23.8%)
Tier of hospital		
Tier 3A	228 (23.1%)	58 (47.5%)
Tier 3 other	302 (30.5%)	38 (31.2%)
Tier 2	370 (37.4%)	19 (15.6%)
Others	89 (9.00%)	7 (5.7%)
Year of experience		
<5	106 (10.7%)	38 (31.2%)
5–10	147 (14.9%)	34 (27.9%)
11–15	195 (19.7%)	27 (22.1%)
>15	541 (54.7%)	23 (18.9%)

The majority of anesthesiologists and nurses held a bachelor's degree, accounting for 75.8% and 72.1%, respectively. Additionally, 71.3% of nurses expressed a desire to pursue further education. We compared staff titles and educational backgrounds among anesthesia medical staff in hospitals of different levels (as shown in Table 2). Our analysis revealed variations in professional titles and academic qualifications among anesthesiologists across hospitals of different tiers. The proportion of senior professional titles and doctoral degrees in tier 3 hospitals was higher than in other hospitals. In contrast, there were no significant differences among nurses across hospitals of varying levels; most held bachelor's degrees, and many had junior professional titles or no professional titles at all.

**Table 2:** The distribution of staff title and education of anesthesiologists and nurses from different tiers of hospitals.

Tier of hospital	Numbers of anesthesiologists	Staff title				Education			
		Ungraded or Junior	Middle	Sub‐senior	Senior	Junior college or below	Bachelor's degree	Master's degree	Doctoral degree
Tier 3A	228	49 (21.5%)	73 (32.0%)	65 (28.5%)	41 (18.0%)	3 (1.3%)	155 (68.0%)	51 (22.4%)	19 (8.3%)
Tier 3 other	302	68 (22.5%)	108 (35.8%)	84 (27.8%)	42 (13.9%)	4 (1.3%)	227 (75.2%)	51 (16.9%)	20 (6.6%)
Tier 2	370	52 (14.1%)	163 (44.1%)	126 (34.1%)	29 (7.8%)	43 (11.6%)	305 (82.4%)	19 (5.1%)	3 (0.8%)
others	89	12 (13.5%)	48 (53.9%)	24 (27.0%)	5 (5.6%)	11 (12.4%)	63 (70.8%)	12 (13.5%)	3 (3.4%)
Total	989	181 (18.3%)	392 (39.6%)	299 (30.2%)	117 (11.8%)	61 (6.2%)	750 (75.8%)	133 (13.5%)	45 (4.6%)
		χ^2^: 39.064	df: 9	*p*＜0.001		χ^2^: 104.683	df: 9	*p*＜0.001	

Tier of hospital	Numbers of nurses	Staff title			Education		
		Ungraded or Junior	Middle	Sub‐senior or Senior	Junior college or below	Bachelor's degree	Master's degree
Tier 3A	58	35 (60.3%)	22 (37.9%)	1 (1.7%)	14 (24.1%)	43 (74.1%)	1 (1.7%)
Tier 3 other	38	23 (60.5%)	14 (36.8%)	1 (2.6%)	14 (36.8%)	24 (63.2%)	0 (0%)
Tier 2	19	10 (52.6%)	9 (47.4%)	0 (0%)	2 (10.5%)	17 (89.5%)	0 (0%)
others	7	4 (57.1%)	3 (42.9%)	0 (0%)	3 (42.9%)	4 (57.1%)	0 (0%)
Total	122	72 (59.0%)	48 (39.3%)	2 (1.6%)	33 (27.1%)	88 (72.1%)	1 (0.8%)
		R:2.234	*p*＞0.05		R:8.263	*p*＞0.05	

### 3.2 Occupational status and job satisfaction

As shown in Table 3, while there were statistical differences between anesthesiologists and nurses regarding the times of night shifts, working hours, number of surgeries involved, and ASA classification ratios, there was no significant difference in perceived workload; most respondents felt that the workload was moderate. Approximately 80.0% of anesthesiologists and nurses reported not participating in teaching activities. Anesthesiologists were more active in scientific research than nurses and published more papers. However, about 70.0% of anesthesiologists did not engage in scientific research and had published no more than five papers.

**Table 3:** The working conditions of participants.

	Total	Anesthesiologist	Nurse	*P*
Times of night shift per month				0.000
<1*	295 (26.6%)	243 (24.6%)	52 (42.6%)	
1–2	132 (11.9%)	118 (11.9%)	14 (11.5%)	
3–4*	192 (17.3%)	180 (18.2%)	12 (9.8%)	
5–6*	257 (23.1%)	238 (24.1%)	19 (15.6%)	
>6	235 (21.2%)	210 (21.2%)	25 (20.5%)	
Working hours per week				0.001
<40	62 (5.6%)	54 (5.5%)	8 (6.6%)	
40–49*	483 (43.5%)	411 (41.6%)	72 (59.0%)	
50–59	327 (29.4%)	296 (29.9%)	31 (25.4%)	
60–69*	125 (11.3%)	118 (11.9%)	7 (5.7%)	
≥70*	114 (10.3%)	110 (11.1%)	4 (3.3%)	
Participated surgeries in the past week				0.000
≤10*	304 (27.4%)	233 (23.6%)	71 (58.2%)	
11–20*	451 (40.6%)	426 (43.1%)	25 (20.5%)	
21–30	185 (16.7%)	172 (17.4%)	13 (10.7%)	
>30	171 (15.4%)	158 (16.0%)	13 (10.7%)	
The proportion of ASA grade Ⅲ or above				0.043
<1/4	626 (56.4%)	562 (56.8%)	64 (52.5%)	
1/4–1/2	261 (23.5%)	238 (24.1%)	23 (18.9%)	
1/2–3/4	140 (12.6%)	121 (12.2%)	19 (15.6%)	
>3/4*	84 (7.6%)	68 (6.9%)	16 (13.1%)	
Heavy workload				0.280
No	73 (6.6%)	64 (6.5%)	9(7.4%)	
Moderate	695 (62.6%)	612 (61.9%)	83(68.0%)	
Yes	343 (30.9%)	313 (31.7%)	30(24.6%)	
Participate in scientific research				0.003
No	783 (70.5%)	683 (69.1%)	100 (82.0%)	
Yes	328 (29.5%)	306(30.9%)	22 (18.0%)	
Total number of published papers				0.000
≤5	827 (74.4%)	710(71.8%)	117 (95.9%)	
>5	284 (25.6%)	279(28.2%)	5 (4.1%)	
Participate in teaching work				0.062
No	882 (79.4%)	793 (80.2%)	89 (73.0%)	
Yes	229 (20.6%)	196 (19.8%)	33 (27.1%)	
Work performance				0.192
Dissatisfied	93 (8.4%)	88 (8.9%)	5 (4.1%)	
Not sure	65 (5.9%)	58 (5.9%)	7 (5.7%)	
Satisfied	953 (85.8%)	843 (85.2%)	110 (90.2%)	
Professional identity				0.019
Identified	986 (88.8%)	870 (88.0%)	116 (95.1%)	
General/Not identified	125 (11.3%)	119 (12.0%)	6 (4.9%)	
Self‐competent identity				0.148
Identified	982 (88.4%)	879 (88.9%)	103 (84.4%)	
General/Not identified	129 (11.6%)	110 (11.1%)	19 (15.6%)	
Minnesota Satisfaction Questionnaire				
Overall job satisfaction	75.57 ± 12.32	74.78 ± 12.18	81.93 ± 11.58	0.000
Intrinsic job satisfaction	46.44 ± 7.15	46.04 ± 7.10	49.68 ± 6.74	0.000
Extrinsic job satisfaction	21.46 ± 4.56	21.16 ± 4.53	23.93 ± 4.05	0.000

* Significant after Bonferroni correction.

Regarding job satisfaction, 85.8% of the staff were satisfied with their work performance, and 88.4% felt competent in their roles, with no significant differences between the two groups in these aspects. However, nurses exhibited a stronger sense of professional identity than anesthesiologists. Similarly, results from the MSQ indicated that nurses scored higher in intrinsic, extrinsic, and overall job satisfaction compared to anesthesiologists.

### 3.3 The stressors encountered by anesthesiology department directors and the existing condition of anesthesiology departments as perceived by their directors

**Figure 2:**
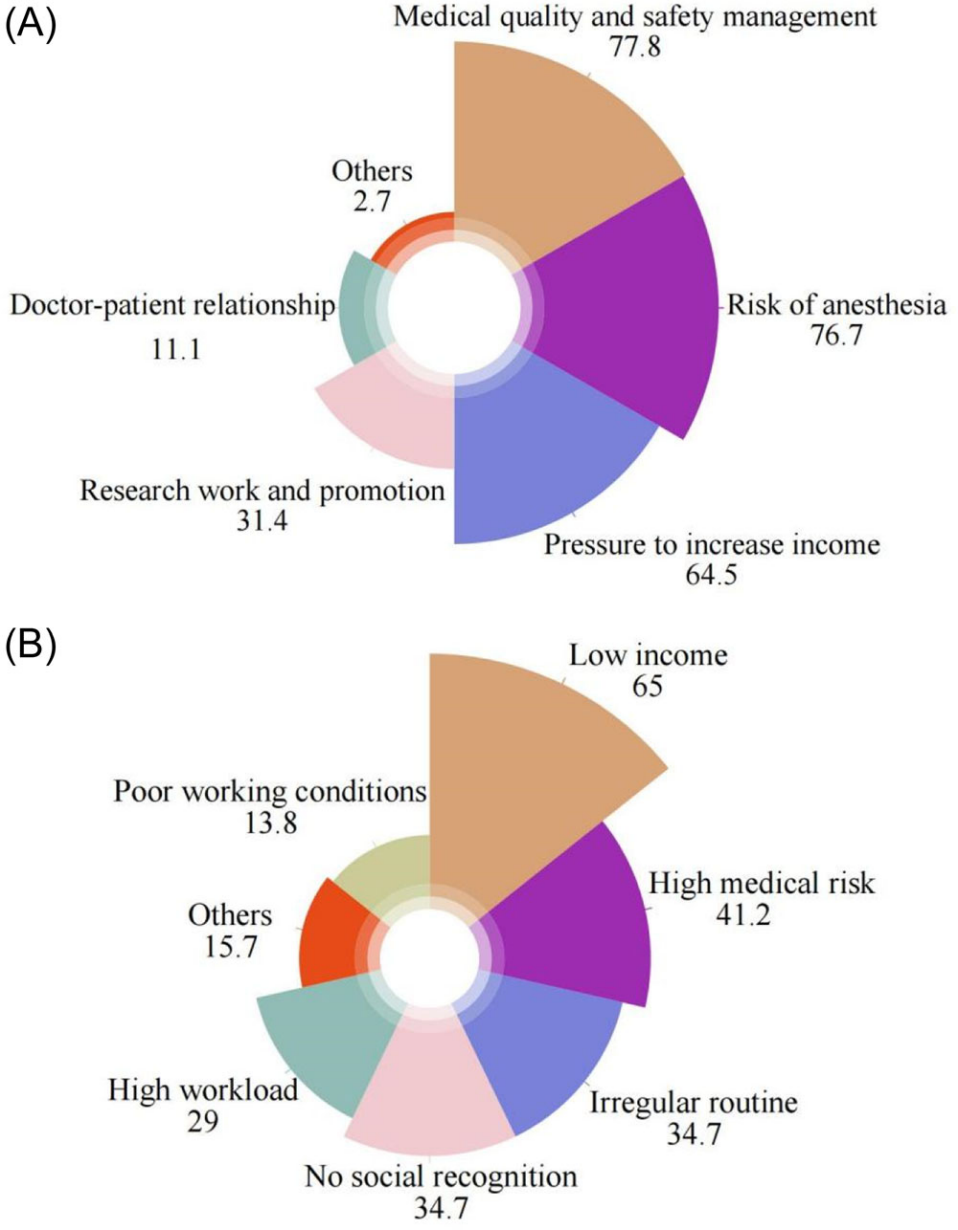
Questionnaire results from the director of anesthesiology departments. (A): Sources of stress for the director of anesthesiology departments. (B): Reasons for employees turnover. Values are all percentages.

A total of 369 anesthesiology department directors participated in the survey. Among these, 194 departments had no more than 5 anesthesiologists, and 326 departments had no more than 5 nurses. Overall, 49.9% of the directors believed that their departments had a staffing shortage problem, and only 29.3% reported no issues with staff excessive fatigue (see Table 4). As shown in Figure 2A, the three major sources of pressure for anesthesiology department directors are medical quality and safety management, anesthesia‐related risks, and increasing income. Figure 2B indicates that the main reason for employee turnover was low income, followed by high medical risks. Irregular schedules and lack of social recognition were tied for the third most significant reasons.

**Table 4:** The current situation of the department according to the Director of anesthesiology.

Grouping	*N* (%)
There are nurses in the department	
Yes	236 (64.0%)
No	133 (36.0%)
Number of anesthesiologists	
≤5	194 (52.6%)
6–10	93 (25.2%)
11–15	28 (7.6%)
16–20	24 (6.5%)
>20	30 (8.1%)
Number of nurses	
≤5	326 (88.4%)
6–10	29 (7.9%)
>10	14 (3.8%)
The total number of workers	
Shortage	184 (49.9%)
Moderate	184 (49.9%)
Overstaffed	1 (0.3%)
Staffs have different degrees of excessive fatigue	
No	108 (29.3%)
Not sure	82 (22.2%)
Yes	179 (48.5%)

### 3.4 Health status

As shown in Table 5, the average SF‐12 scores for both anesthesiologists and nurses were above 95, indicating good physical condition and high quality of life. Although there were no differences in mental health between the two groups, nurses had higher physical health scores compared to anesthesiologists. Most participants reported feeling fatigued a few times per month, with an average MFI‐20 score of 49.10 ± 10.90, indicating moderate fatigue. We divided the participants into two groups: those with severe fatigue (MFI > 60) and those without severe fatigue (MFI ≤ 60). Our findings showed that being aged 31–40, holding a middle title, frequently working night shifts, having long working hours, and participating surgeries classified as ASA III or higher were high‐risk factors for severe fatigue. Individuals with severe fatigue were more likely to perceive their workload as heavy, have lower job satisfaction, experience frequent fatigue, have shorter sleep duration, and be dissatisfied with their sleep quality. See Table 6 for details.

**Table 5:** The health conditions of participants.

	Total	Anesthesiologist	Nurse	*P*
SF‐12 summary	96.97 ± 13.84	96.78 ± 13.85	98.52 ± 13.70	0.158
Physical health	49.70 ± 6.92	49.47 ± 6.94	51.58 ± 6.50	0.001
Mental health	47.27 ± 10.31	47.31 ± 10.34	46.93 ± 10.09	0.617
Feeling tired at work				0.713
Never	120 (10.8%)	106 (10.7%)	14 (11.5%)	
A few times per month	729 (65.6%)	649 (65.6%)	80 (65.6%)	
A few times per week	175 (15.8%)	159 (16.1%)	16 (13.1%)	
Every day	87 (7.8%)	75 (7.6%)	12 (9.8%)	
MFI‐20	49.10 ± 10.90	49.27 ± 10.83	47.72 ± 11.41	0.138
Daily sleep duration				0.005
≤6*	608 (54.7%)	558 (56.4%)	50 (41.0%)	
7*	425 (38.3%)	368 (37.2%)	57 (46.7%)	
≥8*	78 (7.0%)	63 (6.4%)	15 (12.3%)	
Satisfaction of sleep status				0.174
Satisfied	445 (40.1%)	391 (39.5%)	54 (44.3%)	
General	451 (40.6%)	399 (40.3%)	52 (42.6%)	
Dissatisfied	215 (19.4%)	199 (20.1%)	16 (13.1%)	
ISI	8.35 ± 5.32	8.48 ± 5.35	7.24 ± 4.89	0.015

* Significant after Bonferroni correction.

**Table 6:** Comparisons between MFI ≤ 60 and MFI＞60 individuals used chi‐square tests on demographic data and working conditions.

	Total	MFI ≤ 60 (*n* = 966)	MFI＞60 (*n* = 145)	*P*
Gender				0.110
Male	635 (57.2%)	561 (58.1%)	74 (51.0%)	
Female	476 (42.8%)	405 (41.9%)	71 (49.0%)	
Age (years)				<0.001
≤30	185 (16.7%)	164 (17.0%)	21 (14.5%)	
31–40*	397 (35.7%)	320 (33.1%)	77 (53.1%)	
41–50	368 (33.1%)	328 (34.0%)	40 (27.6%)	
>50*	161 (14.5%)	154 (15.9%)	7 (4.8%)	
Job title				0.161
Anesthesiologist	989 (89.0%)	855 (88.5%)	134 (92.4%)	
Nurses	122 (11.0%)	111 (11.5%)	11 (7.6%)	
Marital status				0.138
Single/divorced/widowed	178 (16.0%)	158 (16.4%)	20 (13.8%)	
Married without children	68 (6.1%)	54 (5.6%)	14 (9.7%)	
Married with children	865 (77.9%)	754 (78.1%)	111 (76.6%)	
Education				0.352
Junior college or below	94 (8.5%)	85 (8.8%)	9 (6.2%)	
Bachelor's degree	838 (75.4%)	720 (74.5%)	118 (81.4%)	
Master's degree	134 (12.1%)	120 (12.4%)	14 (9.7%)	
Doctoral degree	45 (4.1%)	41 (4.2%)	4 (2.8%)	
Staff title				<0.001
Ungraded or Junior	253 (22.8%)	224 (23.2%)	29 (20.0%)	
Middle*	440 (39.6%)	362 (37.5%)	78 (53.8%)	
Sub‐senior	300 (27.0%)	266 (27.5%)	34 (23.5%)	
Senior*	118 (10.6%)	114 (11.8%)	4 (2.8%)	
Tier of hospital				0.441
Tier 3A	286 (25.7%)	244 (25.3%)	42 (29.0%)	
Tier 3 other	340 (30.6%)	294 (30.4%)	46 (31.7%)	
Tier 2	389 (35.0%)	340 (35.2%)	49 (33.8%)	
Others	96 (8.6%)	88 (9.1%)	8 (5.5%)	
Times of night shift per month				<0.001
<1*	295 (26.6%)	270 (28.0%)	25 (17.2%)	
1–2*	132 (11.9%)	122 (12.6%)	10 (6.9%)	
3–4	192 (17.3%)	169 (17.5%)	23 (15.9%)	
5–6*	257 (23.1%)	213 (22.1%)	44 (30.3%)	
>6*	235 (21.2%)	192 (19.9%)	43 (29.7%)	
Working hours per week				0.008
<40	62 (5.6%)	56 (5.8%)	6 (4.1%)	
40–49*	483 (43.5%)	438 (45.3%)	45 (31.0%)	
50–59	327 (29.4%)	275 (28.5%)	52 (35.9%)	
60–69*	125 (11.3%)	101 (10.5%)	24 (16.6%)	
≥70	114 (10.3%)	96 (9.9%)	18 (12.4%)	
Participated surgeries in the past week				0.051
≤10*	304 (27.4%)	278 (28.8%)	26 (17.9%)	
11–20	451 (40.6%)	383 (39.7%)	68 (46.9%)	
21–30	185 (16.7%)	157 (16.3%)	28 (19.3%)	
>30	171 (15.4%)	148 (15.3%)	23 (15.9%)	
The proportion of ASA grade Ⅲ or above				0.025
<1/4*	626 (56.4%)	557 (57.7%)	69 (47.6%)	
1/4–1/2*	261 (23.5%)	214 (22.2%)	47 (32.4%)	
1/2–3/4	140 (12.6%)	119 (12.3%)	21 (14.5%)	
>3/4	84 (7.6%)	76 (7.9%)	8 (5.5%)	
Heavy workload				<0.001
No	73 (6.6%)	67 (6.9%)	6 (4.1%)	
Moderate*	695 (62.6%)	629 (65.1%)	66 (45.5%)	
Yes*	343 (30.9%)	270 (28.0%)	73 (50.3%)	
Minnesota Satisfaction Questionnaire				
Overall job satisfaction	75.57 ± 12.32	77.03 ± 11.41	65.84 ± 13.67	<0.001
Intrinsic job satisfaction	46.44 ± 7.15	47.25 ± 6.66	41.01 ± 7.90	<0.001
Extrinsic job satisfaction	21.46 ± 4.56	21.96 ± 4.24	18.12 ± 5.19	<0.001
Feeling tired at work				<0.001
Never*	120 (10.8%)	117 (12.1%)	3 (2.1%)	
A few times per month*	729 (65.6%)	672 (69.6%)	57 (39.3%)	
A few times per week*	175 (15.8%)	131 (13.6%)	44 (30.3%)	
Every day*	87 (7.8%)	46 (4.8%)	41 (28.3%)	
Daily sleep duration				<0.001
≤6*	608 (54.7%)	499 (51.7%)	109 (75.2%)	
7*	425 (38.3%)	392 (40.6%)	33 (22.8%)	
≥8*	78 (7.0%)	75 (7.8%)	3 (2.1%)	
Satisfaction of sleep status				<0.001
Satisfied*	445 (40.1%)	431 (44.6%)	14 (9.7%)	
General	451 (40.6%)	391 (40.5%)	60 (41.4%)	
Dissatisfied*	215 (19.4%)	144 (14.9%)	71 (49.0%)	

* Significant after Bonferroni correction.

**Figure 3:**
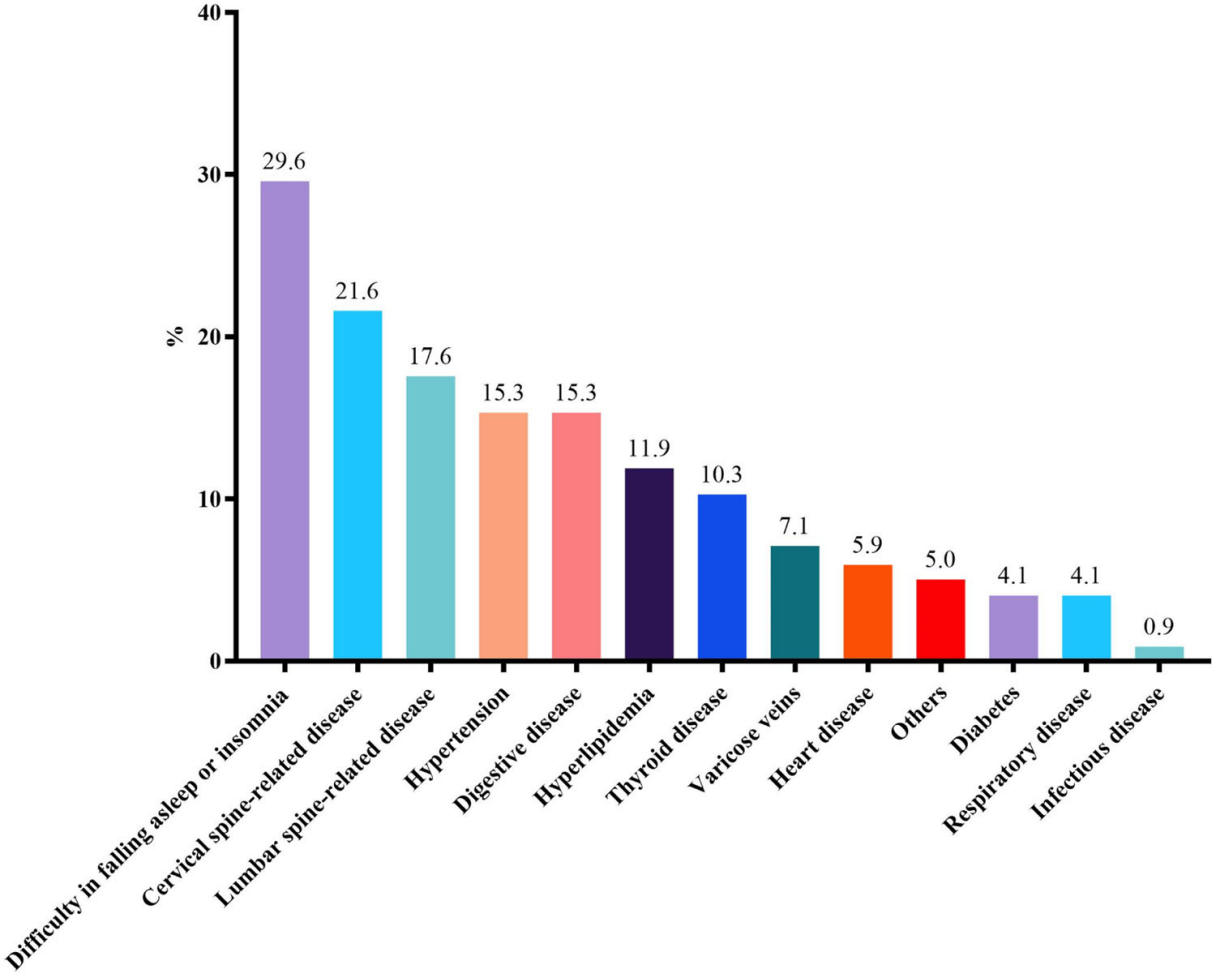
The proportion of different disease distributions among anesthesiologists and nurses.

Most nurses get around 7 h of sleep per day, while anesthesiologists sleep for no more than 6 h. Only 40.1% of the respondents were satisfied with their sleep quality. Similarly, anesthesiologists had higher ISI scores, and there was a statistically significant difference between the two groups (Table 5). The health survey also revealed that difficulty falling asleep/insomnia was the top health issue (Figure 3). We divided the respondents into an insomnia group (ISI ≥ 8) and a non‐insomnia group (ISI < 8)^3^ for multivariate logistic regression analysis (Table 7). The results showed that men (OR = 0.662, 95% CI: 0.482–0.909, *p* < 0.05), those with a bachelor's degree (OR = 2.152, 95% CI: 1.186–3.903, *p* < 0.05), individuals with heavy workloads (OR = 2.999, 95% CI: 1.493–6.024, *p* < 0.01), poor health (OR = 4.280, 95% CI: 1.216–15.057, *p* < 0.05), and high MFI‐20 scores (OR = 1.085, 95% CI: 1.067–1.103, *p* < 0.001) were more likely to suffer from insomnia.

**Table 7:** Multiple binary logistic regression analysis of insomnia‐related factors.

Variable		OR	95% CI	*P*
Gender (male)		0.662	0.482–0.909	0.011
Education	Junior college or below			0.034
	Bachelor's degree	2.152	1.186–3.903	0.012
	Master's degree	1.836	0.844–3.993	0.125
	Doctoral degree	1.135	0.398–3.231	0.813
Heavy workload	No			0.008
	Moderate	2.337	1.238–4.412	0.009
	Yes	2.999	1.493–6.024	0.002
How do you think your health is now	Good			0.000
	General	2.024	1.449–2.828	0.000
	Poor	4.280	1.216–15.057	0.024
MFI‐20		1.085	1.067–1.103	0.000

## 4 DISCUSSION

Nongovernment medical institutions in China are undergoing rapid development, yet the occupational and health status of anesthesia medical staff remains unclear. To the best of our knowledge, the present study represents the largest investigation into the occupational and health status of anesthesiologists and nurses within nongovernment healthcare settings carried out to date. As integral components of the medical and healthcare sector, nongovernment medical institutions share commonalities with their public counterparts while also possessing unique attributes.

### 4.1 Basic information of anesthesia medical staff in nongovernment medical institutions

Examining the distribution of questionnaires across provinces, it was evident that there was a notable disparity in the distribution between the eastern, central, and western regions. While the largest number of questionnaires was gathered from the eastern region, it is important to consider that this distribution might be influenced by questionnaire delivery biases. Another plausible factor could be linked to the varying degrees of regional economic development. Regions that are more advanced economically tend to have well‐established nongovernment medical institutions, which could contribute to a greater likelihood and enthusiasm for participation in surveys conducted by nongovernment medical institution associations.

In nongovernment healthcare institutions at various hospital levels, anesthesiologists with middle staff titles and bachelor's degrees constitute the majority. As the hospital tier increases, the proportion of anesthesiologists with senior professional titles and higher academic degrees also rises. Compared to the national average,^2^ the proportion of personnel with junior college or below is relatively lower, while the proportion of those with a bachelor's degree is higher. This disparity may be related to the department composition and staff numbers. The distribution of staff titles among anesthesiologists in nongovernment hospitals is uneven, with only 18.3% holding ungraded or junior titles, suggesting a shortage of resident doctors in these facilities. This imbalance could stem from nongovernment hospitals' limited appeal to young doctors or from institutional policies that deemphasize the need for resident doctors.

In contrast to anesthesiologists, nurse anesthetists also primarily hold bachelor's degrees. However, most of them have ungraded or junior professional titles, and there is no significant difference in the distribution of professional titles and academic degrees across different hospital tiers. This discrepancy may be influenced by two factors: firstly, the overall number of participating nurses is relatively low, leading to potential bias in the results; secondly, it was not until 2018 that nurse anesthetists were considered specialist nurses^4^ and many hospitals below tier 3 do not have nurse anesthetists. The survey results show that 78.7% of participants come from tier 3 hospitals, and only 64.0% of anesthesiology departments have nursing positions.

### 4.2 Workload and job satisfaction

With the rapid increase in anesthesia cases both inside and outside the operating room, the shortage of anesthesiologists in mainland China remains severe. The annual average workload for each anesthesiologist is still quite high.^5^ This not only leads to fatigue, high burnout, and low job satisfaction, but also reduces the quality of medical care and increases the risk of medical errors.^6^ Previous studies have found that anesthesiologists in China experience heavy workloads, high work stress, high rates of burnout, and low job satisfaction.^7^, ^8^, ^9^ Working hours are a major factor in burnout,^10^ our survey revealed that in nongovernment hospitals, 31.7% of anesthesiologists considered their workload to be heavy. Most anesthesiologists work 40 to 60 h per week and handle no more than 20 surgeries per week, similar to previous findings.^8^ Most were satisfied with their work performance and self‐competent identity, and had a high sense of professional identity. Overall job satisfaction scores were higher than in previous studies.^8^ This may be related to the lower proportion of surgeries classified as ASA III or above, and the limited involvement in research and teaching activities.

Since the proposal by the National Health and Medical Commission in 2017 to establish an anesthesiology nursing unit, the number of nurse anesthetists has been steadily increasing. More recently, the dramatic increases in anesthesiologist workloads associated with the rapidly increasing rate of surgical procedures have increased the need for nurse anesthetists in anesthesiology departments. There is still no unified standard for the responsibilities and authority of nurse anesthetist posts to date. In certain hospitals, nurse anesthetists collaborate with anesthesiologists in tasks such as anesthesia management and vital sign monitoring. In contrast, in other hospitals, nurse anesthetists are not involved in clinical duties such as anesthesia management and post‐anesthesia recovery. Our survey revealed that most nurse anesthetists work less than 50 h per week and are dissatisfied with their income, which is consistent with previous studies.^11^ It is worth noting that our investigation focused solely on the clinical aspects of anesthesia work, which might have contributed to the comparatively lower work intensity experienced by nurses.

### 4.3 Sources of stress for anesthesiology department directors and reasons for employee resignation

Survey results indicate that compared to public hospitals, department directors in anesthesiology at nongovernment hospitals face similar sources of stress,^12^ including anesthesia‐related risks, medical quality and safety management, and the pressure to increase income. The key factors influencing anesthesiologists' resignation differ slightly. Previous studies have shown that low income, heavy workload, and high medical risks were the top three reasons.^12^ However, in nongovernment hospitals, the primary reasons for anesthesiologists' resignation are low income and high medical risks, with irregular working hours and low social recognition tied for third place, and heavy workload ranking only fifth. It is evident that income issues have not significantly improved in recent years. The workload is relatively lower compared to public hospitals, which aligns with the 2022 Statistical Bulletin on the Development of Health Services in China (www.gov.cn).

### 4.4 Excessive fatigue and insomnia

Workplace fatigue has become a prevalent issue, potentially causing negative moods, reduced vigilance, medication errors, and even medical mistakes among anesthesia staff. Our research revealed that 7.8% of medical staff experience daily fatigue at work, while only 11.5% never experience fatigue. The MFI‐20, a commonly used standardized questionnaire, was employed to evaluate the fatigue levels of survey respondents, where a higher total score indicates a greater degree of fatigue.^13^ The results indicated that anesthesiologists generally experience moderate level of fatigue. Variables such as age, staff title, frequency of night shifts, weekly working hours, ASA classification III and above, heavy workload, Minnesota satisfaction score, frequency of fatigue, sleep duration, and satisfaction with sleep emerged as important factors affecting the MFI‐20 scores.

The primary health conditions observed among anesthesiologists and nurses include difficulty falling asleep or insomnia, cervical spine‐related symptoms, and lumbar spine‐related symptoms. These conditions are likely correlated with their work intensity, stress levels, and working environment. Sleep is internationally recognized as a crucial health indicator, and sleep disturbances, particularly insomnia, can contribute to mental health issues.^14^ Highlighting its importance, March 21 is annually recognized as World Sleep Day. Previous studies have indicated that over half of anesthesiologists are dissatisfied with their sleep quality,^8^ and insomnia is prevalent among anesthesiologists.^15^ Consistent with these findings, our research showed that less than half of respondents were satisfied with their sleep quality, and 29.6% experienced difficulties falling asleep. Results from multivariate analysis, which considered both insomnia and non‐insomnia cases, indicated that males, individuals with a bachelor's degree, those experiencing heavy workloads, fatigue, and compromised physical well‐being are more susceptible to insomnia.

Improving the occupational health of healthcare workers can reduce medical errors, enhance their attitude toward care, and improve service quality. Occupational health issues may result in staff resignations or frequent absences, affecting the continuity of care. By improving occupational health and safety (OHS) conditions and reducing the frequency of absences and turnover, patients can receive more stable and consistent care. Physically and mentally healthy medical staff have more energy and motivation to engage in continuing education and professional training, thereby improving their professional skills, which will directly reflect in the quality of care they provide. From a managerial perspective, there is a need to balance the workload of all employees and organize regular stress‐relief activities. From an individual perspective, self‐adjustment, timely relaxation, and seeking professional help when necessary are crucial.

### 4.5 Limitations

Despite our best efforts to enforce quality control measures, the presence of potential information bias could not be entirely eliminated. Firstly, there are no detailed reports on anesthesiologists and nurses in nongovernment hospitals, so we cannot determine the exact proportion of participants, the number of individuals who did not participate, or those who knew the survey but chose not to respond. Secondly, the significant geographical bias among the participants in the questionnaire, which may not be representative of some regions. Additionally, variations in the education and occupational backgrounds of respondents introduced inconsistencies that could result in information bias. Lastly, the influence of the COVID‐19 pandemic brought significant operational and volume changes to hospitals nationwide, and the related negative sentiment may also lead to burnout,^16^, ^17^ which might have led to spurious deviations in questionnaire outcomes.

## 5 CONCLUSION

Our results show that nongovernment medical institutions have fewer employees, similar workloads, relatively low job stress, and higher job satisfaction compared to public hospitals. However, they also face low income and high medical risks, which are significant reasons for turnover. Employees experience moderate overall fatigue, and sleep difficulties and insomnia are health issues that require attention.

The insights obtained from our survey provide important information for the CANMI Anesthesia Committee. These insights should enhance their understanding of the occupational and health status of the workforce and promote the formulation of modern and effective policies. Meanwhile, our research results provide valuable information to readers worldwide. First, it offers a better understanding of the occupational and health status of anesthetic medical staff in nongovernment hospitals in mainland China. Secondly, the situation in some developing countries is similar to that in mainland China, and this study offers a reference for understanding the current situation of anesthesiologists and nurse anesthetists in nongovernment hospitals in certain developing countries. Lastly, by identifying the high‐risk factors for severe fatigue and insomnia, high‐risk personnel within the department can be screened, allowing managers to intervene early.

## AUTHOR CONTRIBUTIONS

WB, LKp and SH are co‐first authors of this paper. Wb and LKp designed the questionnaire，coordinated and supervised data collection and drafted the initial manuscript. SH analysed and interpreted the data, reviewed and revised the manuscript. CXl and QXw edited the manuscript. YL conceptualized and designed the study, designed the questionnaire, mobilized the nongovernment medical staff to actively participate, reviewed and revised the manuscript. SYx, CW and LCh assisted in the questionnaire design, mobilized the nongovernment medical staff to actively participate.

## CONFLICT OF INTEREST STATEMENT

The authors declare no conflicts of interest.

## Data Availability

The data that support the findings of this study are available from the corresponding author upon reasonable request.
